# Explanation

**Published:** 1855-09

**Authors:** 


*Explanation.*—In our article in the August number, in review of the recent transactions of the New York Academy of Medicine, we use the following language, on p. 184, under the head of "Facility of the Operation":

"By some different method of calculation, Dr. Barker throws out a part of the cases as reckoned by the majority, and says that 'Twenty-two patients were subjected to the experiment with the tube.'"

In justice to our friend Dr. Barker, we wish to say to our readers, that this rejection of a part of the cases was confined only to those cases presented at the last meeting of the committee (from the 35th to the 38th inclusive) the record of this meeting not having been furnished to Dr. Barker until after his report was made. The tube was tried in only two of these cases (the 35th and 37th) and in both successfully. It would, therefore, have strengthened Dr. Barker's report had they been included.

We make this explanation not that we suppose any charge of unfairness to be implied in our comment, but because it explains an apparent discrepancy. We may as well add that we do so at our own suggestion and not at the request of any other party.

